# Back to Basics: A Simplified Improvement to Multiple Displacement Amplification for Microbial Single-Cell Genomics

**DOI:** 10.3390/ijms24054270

**Published:** 2023-02-21

**Authors:** Morgan S. Sobol, Anne-Kristin Kaster

**Affiliations:** Institute for Biological Interfaces 5 (IBG-5), Karlsruhe Institute of Technology (KIT), 76344 Eggenstein-Leopoldshafen, Germany

**Keywords:** whole genome amplification, miniaturization, cell sorting, microbial dark matter, contact-free liquid dispenser

## Abstract

Microbial single-cell genomics (SCG) provides access to the genomes of rare and uncultured microorganisms and is a complementary method to metagenomics. Due to the femtogram-levels of DNA in a single microbial cell, sequencing the genome requires whole genome amplification (WGA) as a preliminary step. However, the most common WGA method, multiple displacement amplification (MDA), is known to be costly and biased against specific genomic regions, preventing high-throughput applications and resulting in uneven genome coverage. Thus, obtaining high-quality genomes from many taxa, especially minority members of microbial communities, becomes difficult. Here, we present a volume reduction approach that significantly reduces costs while improving genome coverage and uniformity of DNA amplification products in standard 384-well plates. Our results demonstrate that further volume reduction in specialized and complex setups (e.g., microfluidic chips) is likely unnecessary to obtain higher-quality microbial genomes. This volume reduction method makes SCG more feasible for future studies, thus helping to broaden our knowledge on the diversity and function of understudied and uncharacterized microorganisms in the environment.

## 1. Introduction

The vast majority of bacteria and archaea remain understudied since they have not yet been successfully cultured; thus, their genomes, metabolic potential, and functions in the environment remain unknown [1,2,3,4]. We refer to these microorganisms as microbial dark matter (MDM) [5]. Within MDM hide potentially novel and important solutions for sustainable energy, bioremediation of contaminated environments, and the war against rising antibiotic resistance [6,7,8,9,10]. The use of culture-independent methods to study microorganisms, such as metagenomics, has significantly advanced our understanding of MDM. However, metagenomics still struggles to reliably assemble true, individual genomes due to strain variations and misattribution of sequences to the wrong genomes [11,12]. Furthermore, highly repetitive sequences like those found in CRISPR regions [13,14] are often not accurately assembled and 16S rRNA sequences, as well as mobile genetic elements such as plasmids, are often not attributed to their host organisms [15,16]. As a result, insights into evolutionary mechanisms, like horizontal gene transfer, are lost. Therefore, single-cell genomics (SCG) was developed as a complementary tool to enable the analysis of individual cells, thereby expanding our knowledge of MDM taxa [17,18,19].

**Figure 1:**
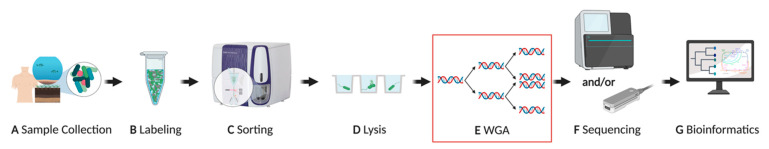
General overview of a single-cell genomics pipeline. (**A**) Environmental samples should be immediately processed, or deep-frozen in the presence of a cryoprotectant that preserves the integrity of the cell. (**B**) Cells are typically stained with a non-specific fluorescent dye, such as DAPI or SYBR^®^ Green, but they can also be specifically labeled, e.g., with fluorescence in situ hybridization. (**C**) Fluorescence activated cell sorting (FACS) is the most common choice for physical isolation of a single cell into multi-well plates (**D**). Once isolated, the single cells are lysed, typically with a combination of alkaline buffer and freeze-thaw cycling, to release the DNA from the cell. (**E**) Whole genome amplification (WGA) is required to generate sufficient amounts of DNA for library preparation since a typical prokaryotic cell only contains a few femtograms of DNA. (**F**) Once DNA libraries are prepared, short- and/or long-read sequencing platforms such as Illumina^®^ and Oxford Nanopore Technologies^®^, respectively, can be employed. (**G**) Finally, bioinformatics is utilized to conduct the quality assessment, assembly, classification, ORF calling, and annotation of the sequences. Created with BioRender.com. Modified from Kaster & Sobol (2020) [17].

**Figure A1:**
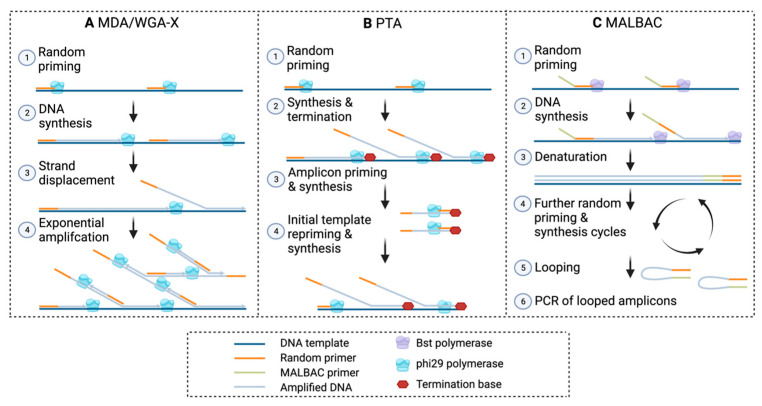
Overview of WGA methods. (**A**) MDA, multiple displacement amplification; WGA-X, 88 whole genome amplification—X. (**B**) PTA, primary template-directed amplification. (**C**) MALBAC, 89 multiple annealing and looping based amplification cycles. Made with Biorender.com.

In general, a microbial SCG workflow (Figure 1) involves (A) sample collection and preservation, (B) specific or non-specific cell staining, (C) cell sorting, (D) cell lysis, (E) whole genome amplification (WGA), and (F,G) genome sequencing and analysis [17,18]. The WGA step is crucial for generating a sufficient amount of input DNA for library preparation and subsequent sequencing, as a typical microbial cell only contains a few femtograms (fg) of DNA [20,21]. Several different WGA methods have been developed and improved upon over the years. These methods can be categorized as polymerase chain reaction (PCR)-based amplification, isothermal amplification, and hybrid, which combines both methods [22]. Pure PCR-based methods, such as degenerate oligonucleotide primed PCR (DOP-PCR) [23], were not successfully applied to microbial single-cells, likely because of sensitivity issues to the low amount of input DNA. The first method to amplify DNA from a single bacterial cell was the so-called multiple displacement amplification (MDA) [24] (Table 1, Appendix A Figure A1-A). MDA is an isothermal method that uses the high-fidelity phi29 polymerase, which has a lower error rate (1 in 10^6^ bases) compared with standard polymerases used in PCR, 3′ → 5′ exonuclease proofreading activity, and generates fragments larger than 10 kb [25,26,27,28] (Table 1). Currently, MDA remains one of the most widely applied methods for amplifying DNA from microbial single cells for these reasons [17].

Unfortunately, MDA also constitutes one of the major limitations in single-cell sequencing due to its high costs (Table 1), as well as its bias against high GC regions, which leads to uneven genome amplification [29,30,31]. Furthermore, artifacts like chimeras and non-specific products can be produced and are thought to occur randomly since sequences that are over-represented in one MDA reaction can be under-represented in another [29,31]. However, some have found these effects to be reproducible due to the fact that a decreased template copy number increases bias and certain sequences are simply not amplified at all [25,29,30,32]. As a result, treatments such as post-amplification endonuclease degradation and post-amplification normalization by nuclease degradation of dsDNA have been used to reduce chimeric sequences [33] and highly abundant sequences [20], respectively.

Other approaches have worked to improve MDA itself, such as WGA-X™, which uses a more thermostable phi29 polymerase for better amplification of high GC organisms [34] (Table 1, Appendix A, Figure A1-A). However, lower genome coverage for organisms with a low GC content compared with standard MDA is reported. More recently, primary template-directed amplification (PTA) was developed, which employs exonuclease-resistant terminators to create smaller amplicons that undergo limited subsequent amplification to limit over-representation of random positions and reduce error propagation [35] (Table 1, Appendix A, Figure A1-B). While this method looks promising to reduce amplification bias, the approach is still in the alpha testing stage for microorganisms (https://www.bioskryb.com/resolvedna-microbiome-alpha/ (accessed on 1 August 2022)) and quite expensive. The hybrid method, multiple annealing and looping based amplification cycles (MALBAC), combines PCR and MDA methods to successfully reduce amplification bias [36,37] (Table 1, Appendix A, Figure A1-C). Yet, MALBAC remains widely unused in microbial SCG, because the Bst and Taq polymerases have higher error rates because they lack proofreading capability [38]. Thus, further work needs to be done to optimize MALBAC, possibly with phi29 or less error-prone enzymes [39].

Even though there is hope to reduce amplification bias in microbial WGA, statistically, inconsistency and bias among the DNA amplification of millions of templates will still persist [41]. In addition, WGA methods are highly sensitive to contamination due to the low amounts of DNA from a single cell. Prior decontamination of reagents with UV [42] can help to remove common reagent contaminants, but this does not prevent other sources of endogenous and/or exogenous contaminants, which become more amplified in larger WGA reaction volumes due to reduced polymerase specificity [43]. Therefore, through bioinformatics, contamination in SAGs needs to be analyzed and removed prior to downstream analysis [12]. Moreover, the large, recommended reaction volumes of these WGA methods also quickly become very costly when applied to high-throughput SCG (Table 1). These high costs limit the depth at which samples can be analyzed, preventing, for example, minority taxa from being captured with SCG.

**Figure 2:**
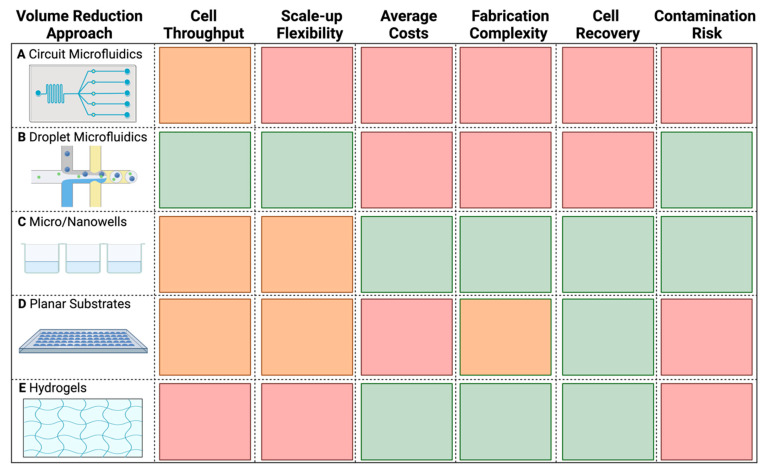
Characteristic comparison of the different microbial SCG volume reduction methods. (**A**) Circuit microfluidics. (**B**) Droplet microfluidics. (**C**) Micro/Nanowells. (**D**) Planar substrates. (**E**) Hydrogels. The color code indicates the relative advantage of a particular approach based on a given feature, from green (better advantage), through orange, to red (less advantage). Table was generated with information from Zhou et al., Fung et al., and Nguyen et al. [54,55,57], as well as from our own experiences with droplet microfluidics, microwells, and planar substrates. Made with Biorender.com.

Therefore, a methodically simpler solution is to reduce WGA’s reaction volume. Reduction of total WGA volume has been shown to increase the concentration of the template and lessens the chance of background contamination being amplified [43]. Furthermore, this approach also significantly reduces the high costs of WGA (Table 1). Previous studies have applied this approach at sub-nanoliter (nL) and picoliter (pL) volumes in microfluidic devices [38,40,44,45,46,47,48], nanowells [49,50], planar surfaces [51,52], and hydrogels [53], which are compared in detail in Figure 2. Many of these approaches and their devices remain largely unused outside of their respective publications, likely because most microfluidic chips and other platforms are not commercially available; they require complex fabrication and operation [54,55], and are therefore hard to access and implement in other research groups. Commercially available options, such as 10× genomics^®^, BD Rhapsody^TM^, and Fluidigm^®^ C1 are costly, less flexible, and geared towards eukaryotic cells. Additionally, current droplet-based technologies sort based on Poisson distributions of cells, resulting in high unoccupancy and low cell recovery [56], which is not applicable for studies analyzing rare populations [57]. Other approaches such as the use of planar substrates require special care to avoid contamination and evaporation, while hydrogel matrices lack the throughput needed for microbial SCG (Figure 2). Hence, the establishment of a reliable and easy-to-use volume reduction method is needed to widen the accessibility and application of microbial SCG.

Surprisingly, there is a lack of information on how bias can be simply reduced between the low- to sub-microliter range within standard 384-well plates. Reduction of standard MDA reaction volumes down to 1.2–2.0 µL have been previously reported [18,58]; however, a systematic assessment of its effect on MDA bias and genome completeness has not yet been done before. Therefore, in this study, we compared the amplification bias in single-amplified genomes (SAGs) of *Escherichia coli* from 10 µL total MDA reaction volumes down to 0.5 µL using novel acoustic liquid dispensing technology developed by Dispendix GmbH (https://www.dispendix.com/ (accessed on 1 August 2022)). Our results indicated that an MDA reaction volume of 1.25 µL is the “sweet-spot” for significantly reducing amplification bias and increasing assembly coverage up to almost 90%, offering an easily accessible approach for future SCG studies to improve WGA in a cost-effective manner.

## 2. Results and Discussion

Previous studies show that volume reduction improves polymerase specificity through “molecular crowding” [59,60]. Molecular crowding reduces competition between amplification of the template and contamination by increasing the probability that polymerase and primers bind to template DNA and reducing spurious binding [59,60]. Moreover, lower reaction volumes reduce the amount of surface area for nonspecific adsorption of nucleic acids to the multi-well plate walls [61,62]. However, too much crowding can also cause adverse effects by causing sterical hinderance and reducing the polymerase from accessing the template [63,64]. Here, we sorted single *E. coli* cells into 384-well plates to compare SAG amplification bias within total MDA microliter and sub-microliter reactions for the first time. In contrast, previous studies have largely examined volume reduction in the sub-nanoliter to picoliter range [38,44,45,47,49,50,51,65].

**Figure 3:**
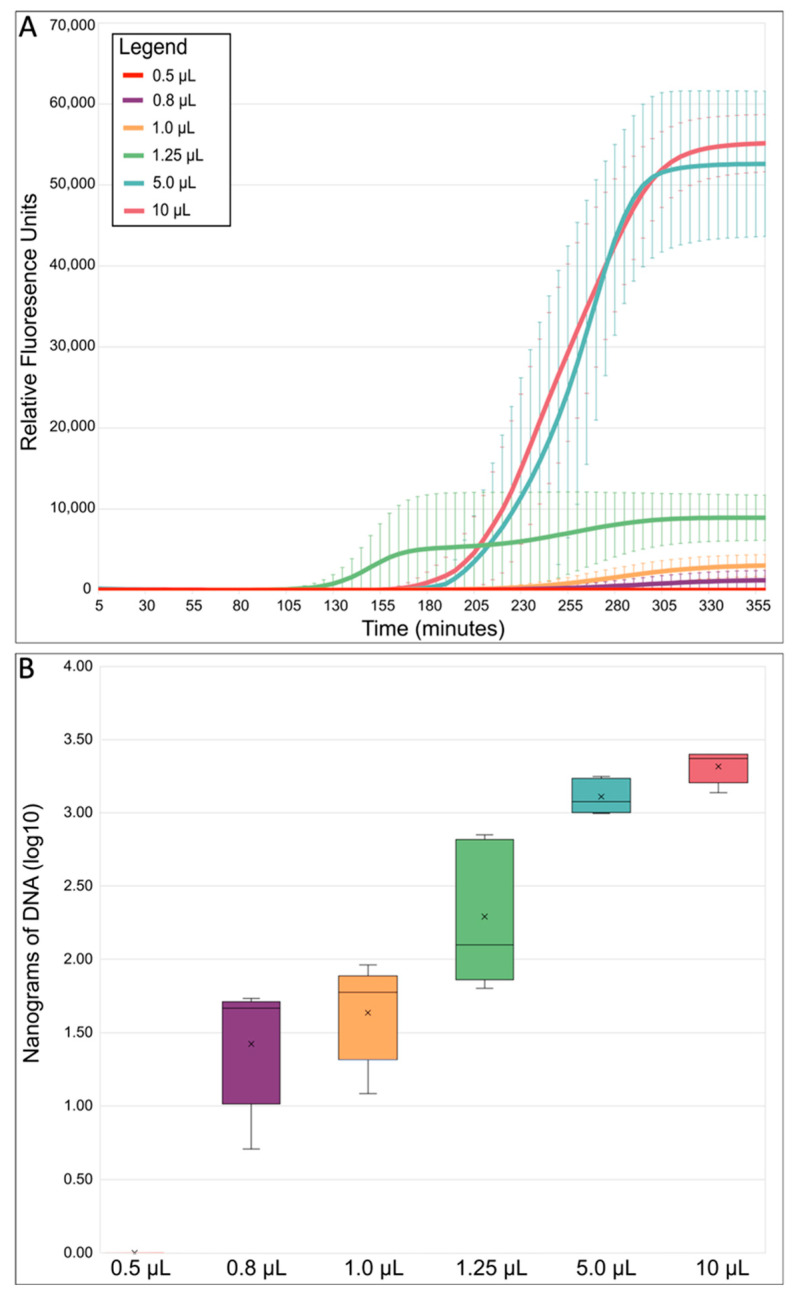
MDA reaction statistics overview. (**A**) Average MDA reaction kinetics by reaction size. Standard error bars represent the standard deviation calculated using all five replicates from each reaction volume. (**B**) Average MDA amplification yield by reaction size. Relative fluorescence units (RFU) refer to the fluorescent signal of SYTO™-13 measured with a real-time thermo-cycler. SYTO™-13 is used to monitor the progression of MDA because it binds to double-stranded DNA as it is amplified. The boxes’ middle line represents the median, and the x represents the mean. Five replicates were used for calculation.

MDAs with total reaction volumes of 0.5, 0.8, 1.0, 1.25, 5.0, and 10 µL were conducted in 384-well plates (Appendix A, Table A1). The smallest-sized MDA reaction, 0.5 µL, did not work and the amplification success rate for the 0.8 and 1.0 µL reactions was only 68.75% and 62.50%, respectively. In comparison, the success rate for the 1.25 µL MDA reactions was 87.50%, whereas both the 5.0 and 10 µL MDA reaction volumes had a success rate of 100%. The lower success rates in the lower MDA reaction volumes was likely due to evaporation and/or sterical hinderance of the polymerase in the small volumes [63,64]. On average, the time that it took for the amplification to reach the detection threshold (indicated as Cq; quantification cycle) was earliest for the 1.25 µL MDA reaction volumes (Figure 3A). Previous studies have reported that earlier Cq values indicated higher genome recovery success and quality [34,66]. Additionally, our detected relative fluorescence (RFU) endpoints and DNA yields from the successful reactions decreased as reaction volumes decreased (Figure 3A,B), initially indicating that volume reduction likely limited the exponential nature of MDA [38,45], which should improve genome coverage and uniformity. To further compare the quality of the WGA reactions, a total of five amplified replicates for each different MDA reaction volume were chosen based on their Cq and RFU values, then subjected to Illumina sequencing using equal amounts of DNA (Appendix A, Table A2).

**Figure 4:**
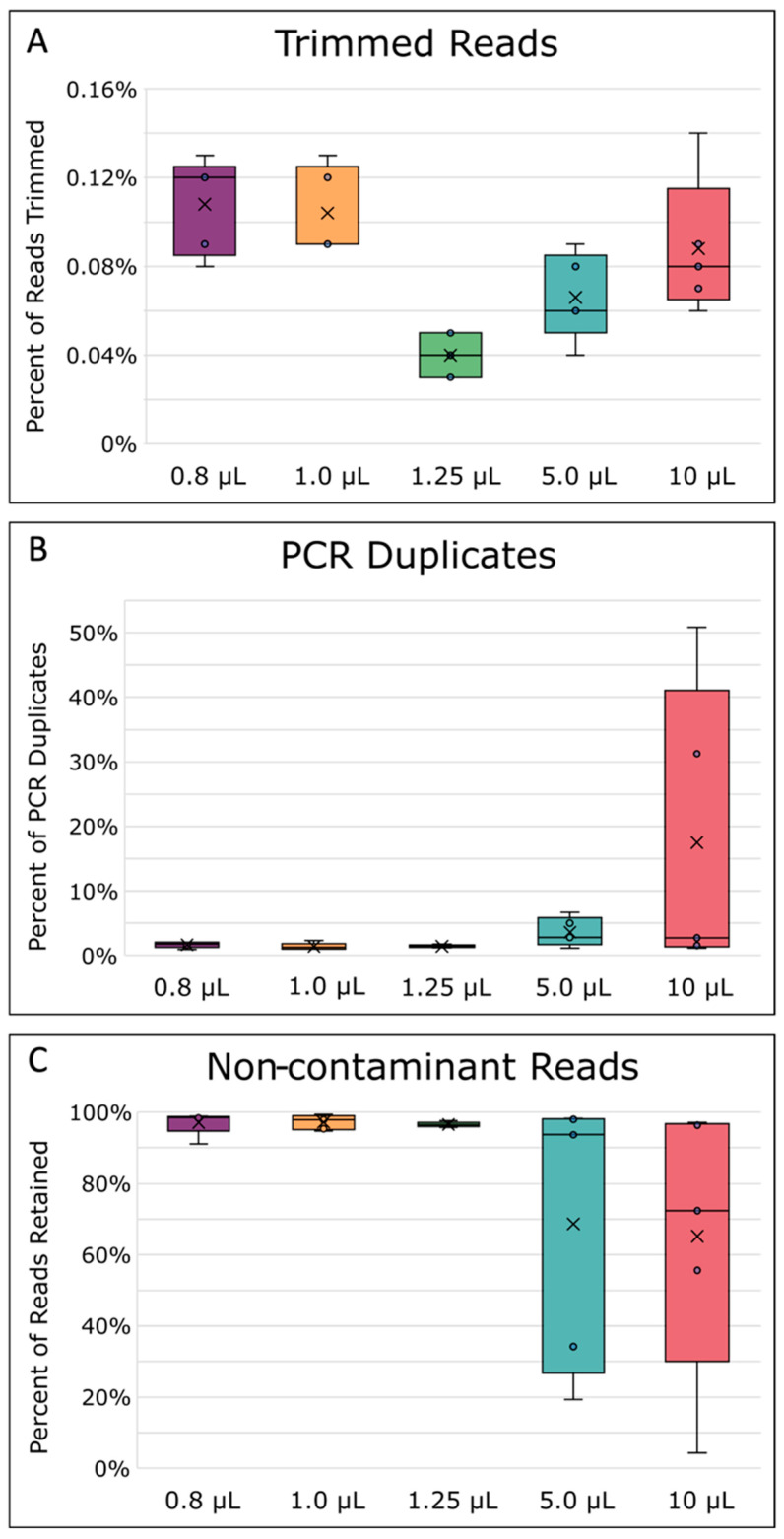
Read processing statistics. (**A**) Percentage of reads removed during quality trimming. (**B**) Percentage of PCR duplicates removed. (**C**) Percentage of reads kept after read contaminant filtering. The boxes’ middle line represents the median, and the x represents the mean. Five replicates were used for calculation.

There was a significant difference in reads lost during read trimming between the different MDA reaction volumes (Figure 4A, *p* = 0.0002 Appendix A, Table A3). On average, the 1.25 µL sized MDA reactions lost significantly fewer reads to read quality trimming compared with all other reaction volumes (*p* ≤ 0.05). After read trimming, all samples were normalized to a 200× sequencing depth before further read processing steps to ensure a fair comparison between the mapping and assembly quality of the different reaction volumes. After depth normalization, the number of duplicated reads was, on average, greater in larger-sized volume reactions (Figure 4B), but the difference between all reactions of the different volumes was not found to be significant (*p* = 0.0870, Appendix A, Table A3). While some amount of read duplication inevitably results from MDA’s exponential amplification nature, comparisons of the percent duplicates between samples could still provide insight into the specificity of the amplification itself. A higher number of duplicates can be caused by the lower template specificity in large MDA reactions causing more spurious priming and amplification [40,51], especially when template concentrations are very low [67]. Furthermore, the issue of lower template specificity also explains why there was an observed trend that larger reaction volumes had more contaminant reads removed after filtering than the smaller reactions (Figure 4C). Lower specificity, leading to more contamination, is likely due to the increased competition between background contamination and the *E. coli* single-cell DNA [40,49]. This increase in contamination was also reflected in the higher amplification gain and product yield mentioned previously (Figure 3A,B), which other studies reported as well [45,47,49]. In general, we also observed that 5 and 10 µL MDA reaction volumes gave less consistent results, as evidenced by larger variation between replicates (Figure 4).

**Figure 5:**
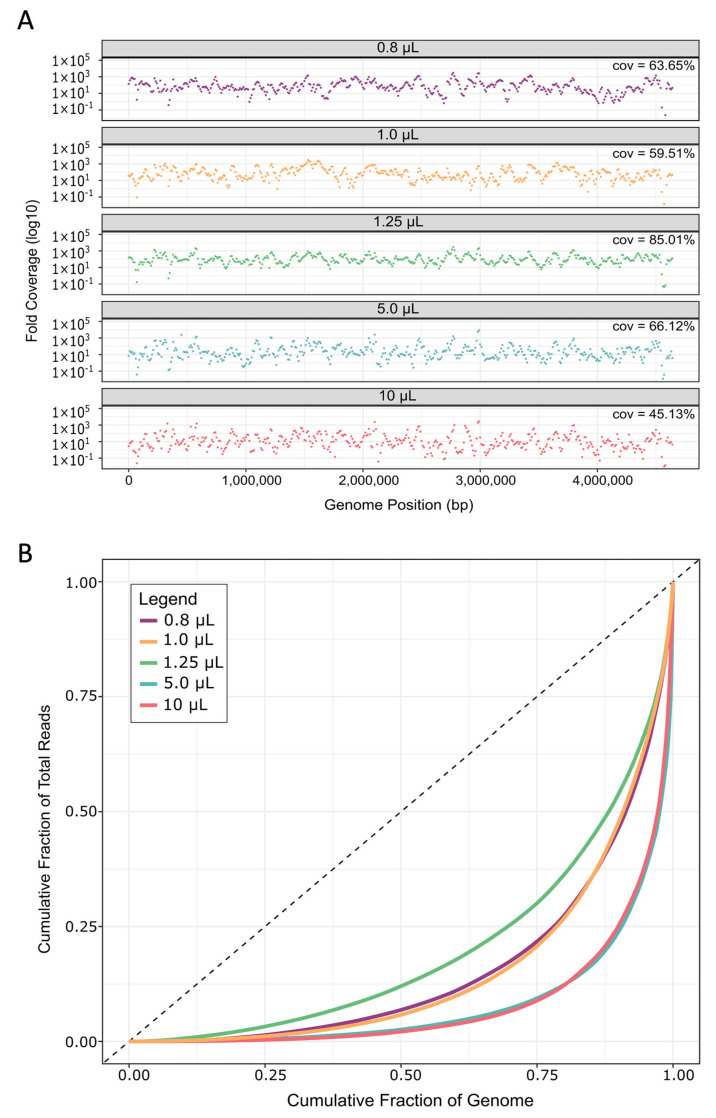
Genome coverage and coverage uniformity bias. (**A**) Read depth from each replicate was calculated in 10 kb bins across the *E. coli* genome. Plots show the average across all replicates for each reaction volume. Cov. is the average coverage breadth, i.e., the percentage of genome positions covered by at least one read. (**B**) Uniformity of read coverage and depth were calculated across 10 kb bins along the *E. coli* genome and averaged for all five replicates of each reaction volume.

As a consequence of lower template specificity, the MDA reaction volumes above 1.25 µL also performed worse during read mapping to the reference *E. coli* MG1655 genome, as indicated by genome coverage breadth and coverage uniformity (Figure 5A,B). MDA in 0.8 and 1.0 µL reaction volumes also resulted in low coverage breadth and uniformity, and a reaction volume of 1.25 µL was therefore determined as the “sweet-spot” for improved MDA in 384-well plates. Likely, the 0.8 and 1.0 µL reaction volumes were simply too low, causing too much molecular crowding, sterically hindering the polymerase from fully accessing the template DNA [63,64], and/or there was too much evaporation. Reduced genome coverage was also recently reported for MDA reaction volumes below 150 nL on a microfluidic system [44], suggesting that platforms independently have a specified “sweet-spot” for efficient MDA.

**Figure A2:**
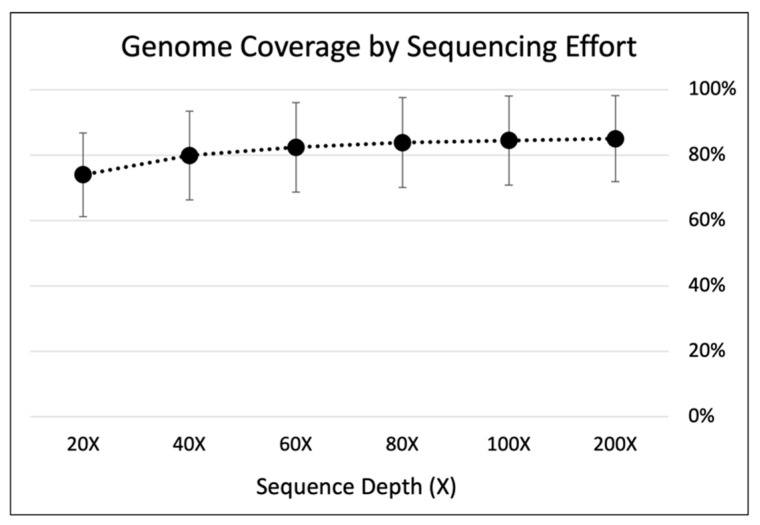
Genome coverage by sequencing effort of MDA in 1.25µL reaction volumes. Trimmed reads were down-sampled from all 1.25 µL samples (*n* = 5) to 200×, 100×, 80×, 60×, 40×, 20× using BBmap reformat.sh [76]. Down-sampled reads were then mapped to *E. coli* MG1655 reference genome with bbmap.sh. Standard error bars were calculated using all five replicates.

On average, reads from 1.25 µL MDA reaction volumes covered 85 ± 13% of the *E. coli* genome, which was 19% to 40% more than the other sized reactions (Figure 5A). This increase in coverage was a large improvement when compared to current, well-established methods like WGA-X™, which gives a reported ~36 ± 21% read coverage of *E. coli* in a standard 10 µL reaction [34]. When compared to 10 µL reactions in this study, we still noted approximately 19% greater coverage breadth than WGA-X™, even though we used ~2 million fewer reads during read mapping. Likely, this difference can be attributed to the lysis modified specifically for *E. coli* herein. Furthermore, the average genome coverage in our study is ~45% greater than MDA performed in ~60 nL hydrogel reactions [53]. Here, the much lower coverage for *E. coli* could be due to the fact that the authors performed a second round of MDA, which has been shown to increase bias [38]. Our reported coverages are also well within range of those reported from a different nanoliter microfluidic method [38], as well as from picoliter droplet reactions [47], at the same sequencing depth (Appendix A, Figure A2). It should be mentioned that one other study reports ~15% greater coverage from MDA in nanoliter microwells when compared with our 1.25 µL average genome coverage at the same sequence depth (20×) (Appendix A, Figure A2) [49]; however, the authors only used three single *E. coli* cells for testing.

To assess the uniformity of read coverage across the genome, reads were averaged into 10 kilo-base (kb) bins and their read depths plotted to visualize coverage depth for each reaction volume (Figure 5A). Especially in the larger volumes, more genome regions are not covered by any reads in comparison to MDA performed in 1.25 µL volumes. Furthermore, coverage depths were more uniform across the genome in 1.25 µL, as evidenced by Lorenz curves [68] showing a more equal distribution of reads covering all bases of the genome (Figure 5B). We further verified this by calculating the Gini index of each sample, which is a measure of deviation from uniformity ranging from 0 (perfectly uniform distribution) to 1 (extremely uneven distribution) [69]. The Gini index differs significantly between different reaction volumes (*p* = 0.0176, Appendix A, Table A4), and is lowest for 1.25 µL reactions (~0.71 ± 0.07, Appendix A, Table A4). These levels of uniformity are similar to those obtained from *E. coli* in 150 nL microfluidic MDA reaction volumes [38] and hydrogels [53].

**Figure 6:**
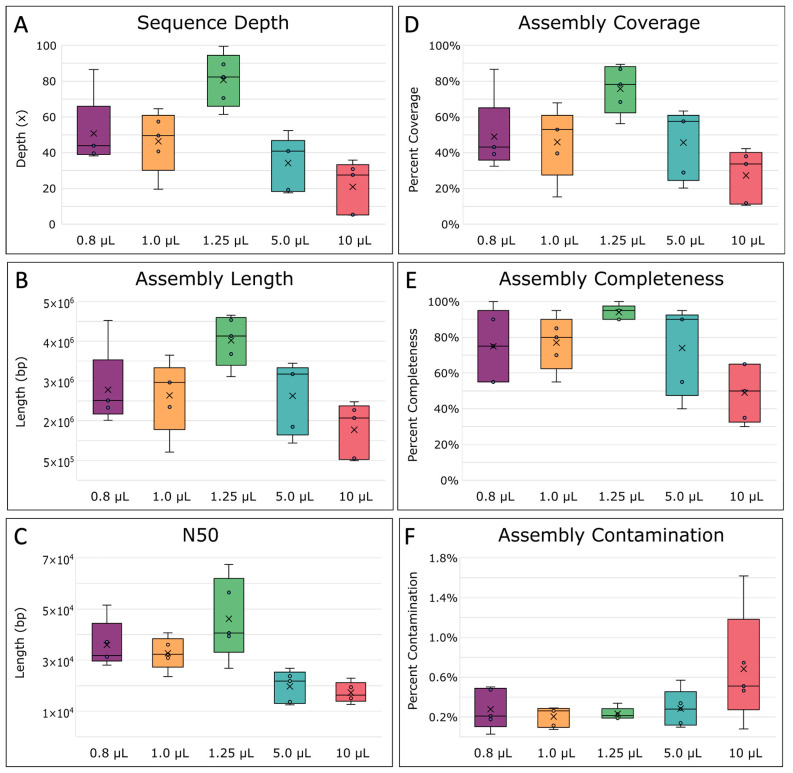
Single-amplified genome (SAG) assembly statistics. (**A**) Final sequence depth, calculated as the estimated number of times each base within the genome was sequenced on average. (**B**) The total average length of the assemblies, (**C**) N50 average, the minimum contig length needed to support 50% of the genome assembly, and (**D**) the percent coverage of the assemblies across the *E. coli* MG1655 reference genome, were all determined with QUAST [70]. (**E**) The completeness of the assembled genome and (**F**) percent of contaminated bases in the assemblies, were determined by MDMCleaner [12]. The boxes’ middle line represents the median, and the x represents the mean. Five replicates were used for calculation.

Next, we assembled and compared SAGs for all replicates. Prior to assembly, read depths were normalized due to the large differences introduced via MDA, setting a target depth of 100×. However, MDA reaction volumes less than and greater than 1.25 µL resulted in lower final sequence depths due to the fact that more reads were lost during the read pre-processing steps (Figure 6A). Therefore, the resulting assemblies were of lower quality compared with assemblies from 1.25 µL MDA reaction volumes (Figure 6B,C). Specifically, 1.25 µL reactions had the longest average total length and N50 at 3,522,851 bp and 46,179 bp, respectively (Figure 6B,C). N50 constitutes the sequence length of the shortest contig representing 50% of the assembly’s total sequence length and indicates that assemblies from 1.25 µL reaction volumes were more contiguous, resulting in higher quality assemblies than the other MDAs. Next, assembly coverage and completeness were calculated. The difference between these two measurements is that coverage is calculated as the percentage of the assembly (contigs) mapped to the reference genome [70], whereas genome completeness was estimated by MDMcleaner as the presence of marker genes such as small subunit (SSU) rRNA genes, large subunit (LSU) rRNA genes, universal bacterial/archaeal protein coding marker genes, total coding sequences (CDS), and tRNA-genes [12]. In general, the assembly coverage (*p* = 0.0199) and completeness (*p* = 0.0128) both significantly differed between the different-sized reactions (Appendix A, Table A5). Not surprisingly, coverage and completeness were highest for assemblies from 1.25 µL MDA reactions and were on average ~75 ± 14% and 94 ± 0.04%, respectively, while contamination was lowest (Figure 6D–F). Three out of five 1.25 µL MDA reaction replicates even achieved over 75% coverage, with the highest being 89.5% (Appendix A, Table A5). Comparatively, WGA-X™ reported *E. coli* assembly coverages of <60%, even with ~5× more reads [34]. Whereas at 10 µL, our assembly coverages were found to be within the range of those reported from WGA-X™ in 10 µL reactions, highlighting how WGA-X™ could also benefit from further volume reduction. In comparison to other volume reduction approaches, our higher assembly coverages were within range of previously reported *E. coli* MDA coverages in pL droplets (88–91%) [47] and nL wells (88–94%) [49] at similar sequence depths.

Overall, these results demonstrate that MDA performed in 1.25 µL reaction volumes greatly improves this method by producing significantly less-biased, less-contaminated, and more complete SAGs than standard, larger reaction volumes. To assess the benefit of further volume reduction, we also tested the 0.5 µL MDA reaction volume in a droplet microarray (DMA) (Aquarray, Germany) since this reaction size did not work in 384-well plates (Figure 3). The DMA is a platform consisting of a glass slide with super-hydrophobic and hydrophilic patterning to create spots in which nanoliter-sized reactions can take place [71,72]. To prevent evaporation during six hours of MDA, the DMA was placed in a humidity chamber [73] and 5% glycerol was added to the MDA master mix. However, these tests were not successful. Recently, the DMA was used to synthesize cDNA from single HeLa cells [73]; however, the cDNA only spent approximately one hour on the DMA versus six hours needed for MDA, and amplification was performed off-chip. Therefore, we attribute our failed MDAs on the DMA to evaporation and/or sterical hinderance of the polymerase [63,64].

Still, further volume reduction could possibly increase genome coverage by ~12–14% [47,49], but the reproducibility of these picoliter and nanoliter approaches is uncertain since few approaches and their results have been validated outside the original study. This is because microfluidic, droplet, and other volume reduction approaches are not as easily accessible or easy to use in other groups, and many are not high-throughput. Additionally, because DNA yield is limited in smaller volumes, some studies have had to perform two rounds of MDA to generate sufficient amounts of products for library preparation [40,53]. However, library preparation input requirements have decreased from ug to pg in the last few years [74], so lower DNA yield is no longer much of an issue.

## 3. Materials and Methods

### 3.1. Bacterial Growth and Isolation

*Escherichia coli* K12 MG1655 (DSMZ 18039) was cultured in 1 mL of Luria Bertani (LB) broth at 30 °C and 750 rpm with the Thermomixer Comfort (Eppendorf, Hamburg, Germany) to the exponential growth phase (~4 h; OD600 of ~2.2–2.6). From this point forward, cells were processed in a UV-decontaminated ISO 4 cleanroom. Equipment and gloves were decontaminated with DNA AWAY (Thermo Fisher Scientific, Waltham, MA, USA). Consumables were UV treated for 1 hr in a Crosslinker and 1 × PBS was UV treated for 6 h in a 254 nm shortwave ultraviolet crosslinker at 0.12 Joules/I^2^ (Analytik Jena GmbH, Jena, Germany).

A BD FACSMelody (Becton-Dickson, Franklin Lakes, NJ, USA), fitted with a 100 µM nozzle and equipped with a 488 nm laser for excitation was used to sort single cells. Cells were first diluted to approximately 10^6^ cells mL^−1^ with sterile 1X PBS to ensure an event rate of <1000 events/s. Gates were defined on side-scatter (cell complexity) and forward-scatter (cell-size). Cells were sorted in single-cell mode into 384-well plates (Bio-Rad, Hercules, CA, USA) containing no sorting buffer (i.e., dry sorting). Plates were sealed with Microseal B (Bio-Rad, Hercules, CA, USA) and stored at −80 °C.

### 3.2. Cell Lysis

Plates containing sorted cells were thawed and centrifuged at 4 °C for 5 min at 3000 rpm (Eppendorf, Germany). Preliminary results found that REPLI-g Single Cell Kit (QIAGEN, Hilden, Germany) lysis buffer was too destructive for *E. coli* single cells; therefore, a modified lysis buffer from Stepanauskas et al. (2017) was used [34]. Cell lysis buffer (0.2 M KOH, 5 mM EDTA and 50 mM DTT) and neutralization buffer (1 M Tris-HCl, pH 4) were treated with UV for 10 min on an ice-water bath in a 254 nm shortwave ultraviolet crosslinker at 0.12 Joules/cm^2^ (Analytik Jena GmbH, Jena, Germany) [42]. The lysis solution was then dispensed onto the cells and into wells containing no cells (negative controls) with an I.DOT mini (Dispendix, Stuttgart, Germany) non-contact liquid dispenser. The plate was incubated at 21 °C for 10 min and neutralized by the addition of an equal volume of neutralization buffer (1 M Tris-HCL, pH 4). The amount of lysis and neutralization buffer per MDA reaction can be found in Appendix A, Table A1.

### 3.3. Multiple Displacement Amplification (MDA)

Multiple displacement amplification (MDA) was performed with the REPLI-g Single Cell Kit (QIAGEN, Hilden, Germany). REPLI-g sc Reaction Buffer and Polymerase were combined in 0.2 mL DNase, RNase-free PCR tubes (Biozym Scientific GmbH, Hessisch Oldendorf, Germany) and UV treated for 30 min on an ice-water bath in a 254 nm shortwave ultraviolet crosslinker at 0.12 Joules/cm^2^ (Analytik Jena GmbH, Jena, Germany) [42]. Syto-13 (Invitrogen, Waltham, NJ, USA) was added to the master mix at a final concentration of 1 µM to monitor exponential DNA amplification. The REPLI-g master mix was then dispensed onto the lysed cells and negative controls with an I.DOT mini (Dispendix, Stuttgart, Germany) non-contact liquid dispenser so that the final MDA volumes were 0.5, 0.8, 1.0, 1.25, 5, and 10 µL. The MDA’s were incubated for 6 h at 30 °C in a CFX-384 thermocycler (Bio-Rad, Hercules, CA, USA), then 65 °C for 10 min to stop the amplification and held at 4 °C. Amplified DNA was kept at −20 °C until used for library preparation.

### 3.4. Library Preparation and Sequencing

The following steps were performed under a UV-decontaminated Laminar Flow PCR workbench (STARLAB International GmbH, Hamburg, Germany), sterilized with DNA AWAY (Thermo Fisher Scientific, Waltham, MA, USA). Prior to library preparation, the amplified DNA was cleaned with DNA Clean & Concentrator—5 (Zymo Research, Irvine, CA, USA). DNA input for library preparation was normalized to 5.98 ng µL^−1^. Libraries were prepared using the NEBNext^®^ Ultra™ II FS DNA Library Prep Kit for Illumina (New England Biolabs (NEB), Ipswich, MA, USA), following the <100 ng input protocol. Fragmentation was set to 14 min and 7 PCR cycles were used. NEBNext^®^ Multiplex Oligos for Illumina^®^ were used for barcoding. Library concentration and size was quantified with Qubit™ DNA HS assay (Life Technologies, Carlsbad, CA, USA) and a Bioanalyzer High Sensitivity DNA kit (Agilent, Santa Clara, CA, USA). The libraries were sequenced using an Illumina NextSeq 550 with the High Output Kit v2.5 300 Cycles (2 × 150 bp paired-end) (Illumina, San Diego, CA, USA).

### 3.5. Data Processing and Analysis

The sequence reads were quality checked using FastQC v0.11.9 (www.bioinformatics.babraham.ac.uk/projects/fastqc (accessed 1 February 2020)) and quality-trimmed using Trim Galore v0.6.6 [75]. Following trimming, reads were normalized to 3,108,153 read pairs (~200× sequence depth) with BBTools v38.87 reformat.sh [76]. Additionally, reads were also down-sampled to 100×, 80×, 60×, 40×, and 20× with reformat.sh to determine the effects of sequence depth on coverage in Appendix A, Figure A2. Normalized reads (200× depth) were assessed for contamination using FASTQ-Screen v0.15.2 [77] against its standard databases for *Homo sapiens*, *Saccharomyces cerevisiae*, *Escherichia coli*, rRNA, phiX, vectors, adapters, as well as *Ralstonia picketti* contaminations. *E. coli* multi-mapping reads were kept. PCR duplicates were counted and removed with dedupe.sh from BBTools [76]. Then, reads were mapped to *E. coli* MG1655 (ASM584v2) with bbmap.sh. Max indel length was set to 80, as recommended for MDA, then coverage was calculated for 10 kb bins [76].

Prior to de novo assembly, the read coverage was normalized with bbnorm.sh setting target = 100 and min = 5 [76]. SPAdes v.3.15.5 was used as recommended for single cells by using the flag –sc for single-cell mode, kmer lengths of 21 to 101 in 10 -step increments, and setting the flag—careful to reduce the number of mismatches [78]. QUAST v.5.2.0 was used to assess assembly quality [70] and MDMcleaner v0.8.3 [12] was used to estimate SAG contamination and completeness. Statistical differences between sample quality, mapping, and assembly statistics were calculated using Anova: Single Factor with an alpha value of 0.05 in Microsoft Excel^®^. For data not normally distributed, as determined by Shapiro–Wilk testing, the non-parametric Kruskal–Wallis one-way ANOVA was used with an alpha value of 0.05. Both the Shapiro–Wilk and Kruskal–Wallis tests were calculated using the Real Statistics Resource Pack software (Release 7.6), Copyright (2013–2021) Charles Zaiontz (www.real-statistics.com (accessed on 1 January 2023)). Pairwise comparisons for measurements with statistical significance were determined using the *t*-Test: two-sample assuming equal variances with an alpha value of 0.05 in Microsoft Excel^®^. Gini indexes were calculated with the ineq package [68] in R v.3.6.3 [79]. Read depth and Lorenz curve plots were created using ggplot2 [80].

## 4. Conclusions

Based on our results, we question whether further volume reduction is really necessary. As reviewed in Figure 2, many of the current nL and pL volume reduction approaches are either too low-throughput, require complex fabrication, and/or are too expensive to make or purchase (>100 USD per device). Therefore, one should gauge for themselves whether the time and cost benefits of volume reduction down to nL and pL reactions make sense in the scope of their study. Meanwhile, volume reduction in standard 384-well plates and with commercially available cell sorters and liquid dispensers makes this approach more easily accessible to other researchers and already drastically reduces the costs by ~97.5% from the standard 50 µL MDA reaction (Table 1). We also found that with our approach, 40× sequence depth is enough for high-quality assemblies (Appendix A, Figure A2), compared to the standard >100× depths generally used in microbial SCG [34,42]. Further cost reduction could also be achieved by applying this approach to the less expensive WGA-X™ method (Table 1), seeing that preliminary work in our group finds WGA-X™ to work in 1.25 µL reaction volumes as well. In the end, we anticipate that the improvements made herein will be of great interest for other single-cell studies and will therefore increase the use of SCG, especially for research focused on elucidating the genomic potential of rare taxa and/or novel microbial dark matter in environmental samples.

## Figures and Tables

**Table 1:** Method characteristics of microbial single-cell genome amplification methods. MDA, multiple displacement amplification; WGA-X, whole genome amplification—X; PTA, primary template-directed amplification; MALBAC, multiple annealing and looping based amplification cycles.

Method Characteristics	Classic MDA ^1–3^	MDA via WGA-X™ ^2^	MDA via PTA ^3,4^	MALBAC ^5^
**Specific Primers**	no	no	no	yes
**Enzyme Type**	phi29	EquiPhi29™	phi29	Bst & Taq polymerase
**Proofreading**	yes	yes	yes	no
**Strand Displacement**	yes	yes	yes	yes
**Amplification Type**	Exponential	Exponential	Quasi-linear	Quasi-linear
**Product Length (nt)**	>10,000	>10,000	250–1500	500–1500
**Average Genome Coverage** **for *E. coli***	~10 to 80%	36 ± 21%	≥92%	~80%
Recommended Reaction Volume *	50 µL	10 µL	20 µL	65 µL
**Approx. Costs per 1.0 µL Reaction**	0.48 $	0.14 $	1.50 $	0.72 $

* Reaction volumes include sorting, lysis, and neutralization buffer volumes, as well as WGA reagents and/or fluorescent dyes to monitor the reaction recommended by the manufacturer or authors of the study. ^1^ [40], ^2^ [34], ^3^ BioSkyrb Genomics, Inc., ^4^ [35], ^5^ [38].

## Data Availability

The sequence reads and genome assemblies generated and analyzed during the current study are available at NCBI GenBank and NCBI SRA under BioProject ID PRJNA900537 (https://www.ncbi.nlm.nih.gov/bioproject/PRJNA900537).

## Appendix A

**Table A1:** MDA reagents by reaction volume.

				Master Mix Reagents		
MDA Reaction Volume	Lysis Buffer	Neutralization Buffer	H_2_O	REPLI-g sc Reaction Buffer	REPLI-g sc DNA Polymerase	Syto-13
0.8 µL	0.056	0.056	0.175	0.464	0.032	0.016
1.0 µL	0.070	0.070	0.219	0.580	0.040	0.020
1.25 µL	0.088	0.088	0.274	0.725	0.050	0.025
5.0 µL	0.350	0.350	1.095	2.900	0.200	0.100
10 µL	0.700	0.700	2.190	5.800	0.400	0.200

**Table A2:** Sample summary. Cq = quantification cycle number, the cycle number at which the fluorescence first rises above the threshold level. RFU = relative fluorescence units.

Sample Name	Cq	RFU Endpoint	Total DNA (ng)
0.8 µL_1	49.15	693	20.896
0.8 µL_2	52.82	432	5.088
0.8 µL_3	45.18	748	48.96
0.8 µL_4	47.39	782	54.08
0.8 µL_5	41.38	3353	46.4
Std Dev	4.2829	1211	21.1098
Average	47.184	1202	35.0848
1.0 µL_1	38.05	4087	59.52
1.0 µL_2	44.91	3315	91.52
1.0 µL_3	44.48	3749	35.2
1.0 µL_4	41.26	3223	64.96
1.0 µL_5	50.8	692	12.13
Std Dev	4.7476	1343	30.2324
Average	43.9	3013	52.6660
1.25 µL_1	34.94	6976	83.25
1.25 µL_2	30.59	6913	125.55
1.25 µL_3	35.49	6695	63.45
1.25 µL_4	22.35	12,238	707.2
1.25 µL_5	24.09	11,769	611.2
Std Dev	6.0633	2823	313.9996
Average	29.492	8918	318.1300
5 µL_1	45.29	45,308	1190.40
5 µL_2	43.56	41,385	1670.40
5 µL_3	42.48	53,918	1772.80
5 µL_4	40.16	61,054	992
5 µL_5	36.7	61,175	1017.60
Std Dev	3.3282	9023	368.5311
Average	41.638	52,568	1328.6400
10 µL_1	34.12	59,245	1369.60
10 µL_2	43.17	52,477	1881.60
10 µL_3	37.06	58,636	2348.80
10 µL_4	42.17	51,944	2515.20
10 µL_5	39.26	52,845	2515.20
Std Dev	3.7068	3591	496.1012
Average	39.156	55,029	2126.0800

**Table A3:** Sample read processing summary. Total reads from all samples were used as input for read trimming in Trim Galore [75]. After read trimming, all samples were down-sampled to 200× sequence depth prior to decontamination with FASTQ-Screen [77] and duplicate removal with dedupe.sh from BBTools [76]. *p*-values were calculated using one-way ANOVA with an alpha of 0.05 in Microscoft Excel^®^.

Sample Name	Reads Lost from Trimming	Non-Contaminated Reads	PCR Duplicates
0.8 µL_1	0.13%	98.55%	1.58%
0.8 µL_2	0.12%	98.38%	2.06%
0.8 µL_3	0.09%	98.84%	1.94%
0.8 µL_4	0.08%	91.11%	1.72%
0.8 µL_5	0.12%	98.51%	0.92%
Std Dev	0.0228%	3.3416%	0.4457%
Average	0.1064%	97.0765%	1.6440%
1.0 µL_1	0.13%	97.89%	1.24%
1.0 µL_2	0.12%	95.35%	0.97%
1.0 µL_3	0.09%	99.44%	2.32%
1.0 µL_4	0.09%	94.75%	0.97%
1.0 µL_5	0.09%	98.50%	1.37%
Std Dev	0.0192%	2.0355%	0.5566%
Average	0.1047%	97.1864%	1.3740%
1.25 µL_1	0.03%	96.52%	1.41%
1.25 µL_2	0.03%	96.26%	1.43%
1.25 µL_3	0.04%	95.74%	1.75%
1.25 µL_4	0.05%	97.57%	1.28%
1.25 µL_5	0.05%	96.60%	1.22%
Std Dev	0.0086%	0.6685%	0.2054%
Average	0.0396%	96.5391%	1.4180%
5 µL_1	0.08%	93.65%	2.79%
5 µL_2	0.06%	98.25%	6.65%
5 µL_3	0.04%	98.03%	5.01%
5 µL_4	0.06%	19.33%	1.11%
5 µL_5	0.09%	34.24%	2.31%
Std Dev	0.0188%	38.6685%	2.2253%
Average	0.0665%	68.6991%	3.5740%
10 µL_1	0.08%	55.66%	1.55%
10 µL_2	0.09%	96.40%	50.82%
10 µL_3	0.14%	72.44%	2.71%
10 µL_4	0.06%	97.09%	31.26%
10 µL_5	0.07%	4.37%	1.16%
Std Dev	0.0292%	38.1900%	22.5815%
Average	0.0887%	65.1911%	17.5000%
*p*-value	0.0002	0.0917	0.0870

**Table A4:** MDA read mapping summary. After contaminant and duplicate removal, reads were mapped to *E. coli* MG1655 reference genome with bbmap.sh [76]. Mapping statistics were also calculated with BBmap. Gini indices were calculated with the ineq package [68] in R studio [79]. *p*-values were calculated using one-way ANOVA with an alpha of 0.05 in Microsoft Excel^®^.

Sample Name	Mapping Coverage %	Avg. Insert Size	Read Std dev/10 Kb	Gini Index
0.8 µL_1	59.46	223.03	572.965	0.8708
0.8 µL_2	58.17	211.67	750.424	0.9022
0.8 µL_3	58.13	209.02	664.428	0.8836
0.8 µL_4	47.45	212.57	578.596	0.8907
0.8 µL_5	95.03	239.46	380.321	0.6733
Std Dev	18.2003	12.5441	137.5307	0.0962
Average	63.6494	219.1500	589.3468	0.8441
1.0 µL_1	69.73	231.81	598.19	0.8570
1.0 µL_2	85.01	231.36	459.222	0.7783
1.0 µL_3	21.20	212.69	927.816	0.9557
1.0 µL_4	67.73	226.17	417.53	0.8096
1.0 µL_5	53.86	226.44	653.862	0.8913
Std Dev	24.0911	7.7362	201.7674	0.0696
Average	59.5071	225.6940	611.3240	0.8584
1.25 µL_1	90.63	200.66	299.296	0.6762
1.25 µL_2	96.14	195.93	304.19	0.6360
1.25 µL_3	78.95	213.10	471.041	0.7786
1.25 µL_4	94.53	241.56	513.246	0.6831
1.25 µL_5	64.79	237.90	490.337	0.7967
Std Dev	13.1510	20.9923	105.039	0.0698
Average	85.0079	217.8300	415.6220	0.7141
5 µL_1	85.73	216.86	977.542	0.8520
5 µL_2	81.74	223	1179.344	0.8868
5 µL_3	82.06	235	1192.636	0.8862
5 µL_4	48.39	212.43	175.801	0.8889
5 µL_5	32.67	201.55	451.882	0.9372
Std Dev	24.0625	12.4163	458.3374	0.0304
Average	66.1188	217.7680	795.4410	0.8902
10 µL_1	61.45	224.95	385.936	0.8604
10 µL_2	24.69	255.58	714.113	0.9761
10 µL_3	62.34	219.01	862.061	0.9161
10 µL_4	53.45	211.13	818.291	0.9153
10 µL_5	23.72	238.46	71.785	0.9341
Std Dev	19.4160	17.5222	335.3406	0.0106
Average	45.1300	229.8260	570.4372	0.9204
*p*-value	0.0939	0.6265	0.3561	0.0176

**Table A5:** MDA assembly summary. After read processing, the final sequence depth used for assembly was calculated. Assembly N50, length, and coverage were calculated using QUAST [70]. Coverage is calculated as the percent of contigs aligned to the reference genome. MDMcleaner was used to calculate completeness and assembly contamination [12]. *p*-values were calculated using one-way ANOVA with an alpha of 0.05 in Microscoft Excel^®^.

Sample Name	Final Sequence Depth (×)	N50	Length (bp)	Genome Coverage	Genome Completeness	Fraction of Untrusted Base-Pairs
0.8 µL_1	45	37,203	2,008,434	43.18%	90%	0.21%
0.8 µL_2	40	31,827	1,826,589	39.29%	55%	0.50%
0.8 µL_3	44	28,055	2,033,477	43.71%	55%	0.03%
0.8 µL_4	38	31,357	1,508,083	32.39%	75%	0.48%
0.8 µL_5	87	51,543	4,023,801	86.59%	100%	0.18%
Std Dev	20	9289	997,096	21.48%	20%	0.20%
Average	51	35,997	2,280,077	49.03%	75%	0.28%
1.0 µL_1	50	30,920	2,510,486	54.00%	85%	0.26%
1.0 µL_2	65	36,080	3,151,225	67.81%	95%	0.29%
1.0 µL_3	20	40,727	711,913	15.32%	55%	0.11%
1.0 µL_4	57	32,298	2,462,399	52.94%	80%	0.08%
1.0 µL_5	41	23,582	1,846,315	39.66%	70%	0.28%
Std Dev	17	6370	920,516	19.81%	15%	0.10%
Average	46	32,721	2,136,468	45.94%	77%	0.21%
1.25 µL_1	89	56,529	3,630,701	78.16%	95%	0.23%
1.25 µL_2	100	67,482	4,156,954	89.46%	95%	0.21%
1.25 µL_3	71	39,389	3,179,043	68.40%	90%	0.34%
1.25 µL_4	82	26,847	4,034,821	86.81%	100%	0.19%
1.25 µL_5	61	40,650	2,612,738	56.20%	90%	0.19%
Std Dev	15	15,901	636,856	13.71%	4%	0.06%
Average	81	46,179	3,522,851	75.81%	94%	0.23%
5 µL_1	52	26,847	2,945,211	63.31%	95%	0.28%
5 µL_2	41	21,803	2,673,880	57.49%	90%	0.14%
5 µL_3	41	23,782	2,720,379	58.46%	90%	0.10%
5 µL_4	19	13,693	1,347,172	28.91%	55%	0.57%
5 µL_5	18	12,525	940,124	20.19%	40%	0.34%
Std Dev	15	6319	913,433	19.65%	25%	0.19%
Average	34	19,730	2,125,353	45.67%	74%	0.29%
10 µL_1	36	22,962	1,973,202	42.25%	65%	0.47%
10 µL_2	5	15,114	494,824	10.64%	30%	1.62%
10 µL_3	31	19,415	1,768,861	37.97%	65%	0.75%
10 µL_4	28	16,327	1,568,571	33.69%	50%	0.51%
10 µL_5	5	12,760	550,195	11.82%	35%	0.08%
Std Dev	14	3964	698,482	14.96%	16%	0.57%
Average	21	17,316	1,271,131	27.27%	49%	0.68%
*p*-value	0.0043	0.0004	0.0199	0.0199	0.0128	0.4077

## References

[1] Wu D., Raymond J., Wu M., Chatterji S., Ren Q., Graham J.E., Bryant D.A., Robb F., Colman A., Tallon L.J. (2009). Complete Genome Sequence of the Aerobic CO-Oxidizing Thermophile *Thermomicrobium Roseum*. PLoS ONE.

[2] McDonald D., Price M.N., Goodrich J., Nawrocki E.P., Desantis T.Z., Probst A., Andersen G.L., Knight R., Hugenholtz P. (2012). An Improved Greengenes Taxonomy with Explicit Ranks for Ecological and Evolutionary Analyses of Bacteria and Archaea. ISME J..

[3] Hug L.A., Baker B.J., Anantharaman K., Brown C.T., Probst A.J., Castelle C.J., Butterfield C.N., Hernsdorf A.W., Amano Y., Ise K. (2016). A New View of the Tree of Life. Nat. Microbiol..

[4] Lloyd K.G., Steen A.D., Ladau J., Yin J., Crosby L. (2018). Phylogenetically Novel Uncultured Microbial Cells Dominate Earth Microbiomes. mSystems.

[5] Solden L., Lloyd K., Wrighton K. (2016). The Bright Side of Microbial Dark Matter: Lessons Learned from the Uncultivated Majority. Curr. Opin. Microbiol..

[6] Abou Seeda M.A., Yassen A.A., Abou El-Nour E.Z.A.A. (2017). Microorganism as a Tool of Bioremediation Technology for Cleaning Waste and Industrial Water. Biosci. Res..

[7] Katz L., Baltz R.H. (2016). Natural Product Discovery: Past, Present, and Future. J. Ind. Microbiol. Biotechnol..

[8] Kumar R., Kumar P. (2017). Future Microbial Applications for Bioenergy Production: A Perspective. Front. Microbiol..

[9] Mullis M.M., Rambo I.M., Baker B.J., Reese B.K. (2019). Diversity, Ecology, and Prevalence of Antimicrobials in Nature. Front. Microbiol..

[10] Stincone P., Brandelli A. (2020). Marine Bacteria as Source of Antimicrobial Compounds. Crit. Rev. Biotechnol..

[11] Dick G.J., Andersson A.F., Baker B.J., Simmons S.L., Thomas B.C., Yelton A.P., Banfield J.F. (2009). Community-Wide Analysis of Microbial Genome Sequence Signatures. Genome Biol..

[12] Vollmers J., Wiegand S., Lenk F., Kaster A.-K. (2022). How Clear Is Our Current View on Microbial Dark Matter? (Re-)Assessing Public MAG & SAG Datasets with MDMcleaner. Nucleic Acids Res..

[13] Acuña-Amador L., Primot A., Cadieu E., Roulet A., Barloy-Hubler F. (2018). Genomic Repeats, Misassembly and Reannotation: A Case Study with Long-Read Resequencing of *Porphyromonas gingivalis* Reference Strains. BMC Genom..

[14] Skennerton C.T., Imelfort M., Tyson G.W. (2013). Crass: Identification and Reconstruction of CRISPR from Unassembled Metagenomic Data. Nucleic Acids Res..

[15] Dam H.T., Vollmers J., Sobol M.S., Cabezas A., Kaster A.K. (2020). Targeted Cell Sorting Combined With Single Cell Genomics Captures Low Abundant Microbial Dark Matter With Higher Sensitivity Than Metagenomics. Front. Microbiol..

[16] Maguire F., Jia B., Gray K.L., Yin Venus Lau W., Beiko R.G., Brinkman F.S.L. (2020). Metagenome-Assembled Genome Binning Methods with Short Reads Disproportionately Fail for Plasmids and Genomic Islands. Microb. Genom..

[17] Kaster A.K., Sobol M.S. (2020). Microbial Single-Cell Omics: The Crux of the Matter. Appl. Microbiol. Biotechnol..

[18] Rinke C., Lee J., Nath N., Goudeau D., Thompson B., Poulton N., Dmitrieff E., Malmstrom R., Stepanauskas R., Woyke T. (2014). Obtaining Genomes from Uncultivated Environmental Microorganisms Using FACS-Based Single-Cell Genomics. Nat. Protoc..

[19] Stepanauskas R. (2012). Single Cell Genomics: An Individual Look at Microbes. Curr. Opin. Microbiol..

[20] Rodrigue S., Malmstrom R.R., Berlin A.M., Birren B.W., Henn M.R., Chisholm S.W. (2009). Whole Genome Amplification and De Novo Assembly of Single Bacterial Cells. PLoS ONE.

[21] Hedlund B.P., Dodsworth J.A., Murugapiran S.K., Rinke C., Woyke T. (2014). Impact of Single-Cell Genomics and Metagenomics on the Emerging View of Extremophile “Microbial Dark Matter”. Extremophiles.

[22] Gawad C., Koh W., Quake S.R. (2016). Single-Cell Genome Sequencing: Current State of the Science. Nat. Rev. Genet..

[23] Telenius H., Carter N.P., Bebb C.E., Nordenskjöld M., Ponder B.A.J., Tunnacliffe A. (1992). Degenerate Oligonucleotide-Primed PCR: General Amplification of Target DNA by a Single Degenerate Primer. Genomics.

[24] Raghunathan A., Ferguson H.R., Bornarth C.J., Song W., Driscoll M., Lasken R.S. (2005). Genomic DNA Amplification from a Single Bacterium. Appl. Environ. Microbiol..

[25] Dean F.B., Nelson J.R., Giesler T.L., Lasken R.S. (2001). Rapid Amplification of Plasmid and Phage DNA Using Phi29 DNA Polymerase and Multiply-Primed Rolling Circle Amplification. Genome Res..

[26] Zhang D.Y., Brandwein M., Hsuih T., Li H.B. (2001). Ramification Amplification: A Novel Isothermal DNA Amplification Method. Mol. Diagn..

[27] Esteban J.A., Salas M., Blanco L. (1993). Fidelity of Φ29 DNA Polymerase. Comparison between Protein-Primed Initiation and DNA Polymerization. J. Biol. Chem..

[28] Paez J.G., Lin M., Beroukhim R., Lee J.C., Zhao X., Richter D.J., Gabriel S., Herman P., Sasaki H., Altshuler D. (2004). Genome Coverage and Sequence Fidelity of Phi29 Polymerase-Based Multiple Strand Displacement Whole Genome Amplification. Nucleic Acids Res..

[29] Lasken R.S., Stockwell T.B. (2007). Mechanism of Chimera Formation during the Multiple Displacement Amplification Reaction. BMC Biotechnol..

[30] Lasken R.S. (2009). Genomic DNA Amplification by the Multiple Displacement Amplification (MDA) Method. Biochem. Soc. Trans..

[31] Sabina J., Leamon J.H., Kroneis T. (2015). Bias in Whole Genome Amplification: Causes and Considerations. Methods in Molecular Biology.

[32] Wu L., Liu X., Schadt C.W., Zhou J. (2006). Microarray-Based Analysis of Subnanogram Quantities of Microbial Community DNAs by Using Whole-Community Genome Amplification. Appl. Environ. Microbiol..

[33] Zhang K., Martiny A.C., Reppas N.B., Barry K.W., Malek J., Chisholm S.W., Church G.M. (2006). Sequencing Genomes from Single Cells by Polymerase Cloning. Nat. Biotechnol..

[34] Stepanauskas R., Fergusson E.A., Brown J., Poulton N.J., Tupper B., Labonté J.M., Becraft E.D., Brown J.M., Pachiadaki M.G., Povilaitis T. (2017). Improved Genome Recovery and Integrated Cell-Size Analyses of Individual Uncultured Microbial Cells and Viral Particles. Nat. Commun..

[35] Gonzalez-Pena V., Natarajan S., Xia Y., Klein D., Carter R., Pang Y., Shaner B., Annu K., Putnam D., Chen W. (2021). Accurate Genomic Variant Detection in Single Cells with Primary Template-Directed Amplification. Proc. Natl. Acad. Sci. USA.

[36] Lu S., Zong C., Fan W., Yang M., Li J., Chapman A.R., Zhu P., Hu X., Xu L., Yan L. (2012). Probing Meiotic Recombination and Aneuploidy of Single Sperm Cells by Whole-Genome Sequencing. Science.

[37] Zong C., Lu S., Chapman A.R., Xie X.S. (2012). Genome-Wide Detection of Single-Nucleotide and Copy-Number Variations of a Single Human Cell. Science.

[38] De Bourcy C.F.A., De Vlaminck I., Kanbar J.N., Wang J., Gawad C., Quake S.R. (2014). A Quantitative Comparison of Single-Cell Whole Genome Amplification Methods. PLoS ONE.

[39] Lasken R.S. (2013). Single-Cell Sequencing in Its Prime. Nat. Biotechnol..

[40] Marcy Y., Ishoey T., Lasken R.S., Stockwell T.B., Walenz B.P., Halpern A.L., Beeson K.Y., Goldberg S.M.D., Quake S.R. (2007). Nanoliter Reactors Improve Multiple Displacement Amplification of Genomes from Single Cells. PLoS Genet..

[41] Zhang C.-Z., Adalsteinsson V.A., Francis J., Cornils H., Jung J., Maire C., Ligon K.L., Meyerson M., Love J.C., Author N.C. (2016). Calibrating Genomic and Allelic Coverage Bias in Single-Cell Sequencing. HHS Public Access Author Manuscript. Nat. Commun..

[42] Woyke T., Sczyrba A., Lee J., Rinke C., Tighe D., Clingenpeel S., Malmstrom R., Stepanauskas R., Cheng J.-F. (2011). Decontamination of MDA Reagents for Single Cell Whole Genome Amplification. PLoS ONE.

[43] Hutchison C.A., Smith H.O., Pfannkoch C., Venter J.C. (2005). Cell-Free Cloning Using Φ29 DNA Polymerase. Proc. Natl. Acad. Sci. USA.

[44] Ruan Q., Ruan W., Lin X., Wang Y., Zou F., Zhou L., Zhu Z., Yang C. (2020). Digital-WGS: Automated, Highly Efficient Whole-Genome Sequencing of Single Cells by Digital Microfluidics. Sci. Adv..

[45] Rhee M., Light Y.K., Meagher R.J., Singh A.K. (2016). Digital Droplet Multiple Displacement Amplification (ddMDA) for Whole Genome Sequencing of Limited DNA Samples. PLoS ONE.

[46] Sidore A.M., Lan F., Lim S.W., Abate A.R. (2015). Enhanced Sequencing Coverage with Digital Droplet Multiple Displacement Amplification. Nucleic Acids Res..

[47] Nishikawa Y., Hosokawa M., Maruyama T., Yamagishi K., Mori T., Takeyama H. (2015). Monodisperse Picoliter Droplets for Low-Bias and Contamination-Free Reactions in Single-Cell Whole Genome Amplification. PLoS ONE.

[48] Blainey P.C., Mosier A.C., Potanina A., Francis C.A., Quake S.R. (2011). Genome of a Low-Salinity Ammonia-Oxidizing Archaeon Determined by Single-Cell and Metagenomic Analysis. PLoS ONE.

[49] Gole J., Gore A., Richards A., Chiu Y.J., Fung H.L., Bushman D., Chiang H.I., Chun J., Lo Y.H., Zhang K. (2013). Massively Parallel Polymerase Cloning and Genome Sequencing of Single Cells Using Nanoliter Microwells. Nat. Biotechnol..

[50] Goldstein L.D., Chen Y.J.J., Dunne J., Mir A., Hubschle H., Guillory J., Yuan W., Zhang J., Stinson J., Jaiswal B. (2017). Massively Parallel Nanowell-Based Single-Cell Gene Expression Profiling. BMC Genom..

[51] Leung K., Klaus A., Lin B.K., Laks E., Biele J., Lai D., Bashashati A., Huang Y.-F.F., Aniba R., Moksa M. (2016). Robust High-Performance Nanoliter-Volume Single-Cell Multiple Displacement Amplification on Planar Substrates. Proc. Natl. Acad. Sci. USA.

[52] Rezaei M., Radfar P., Winter M., McClements L., Thierry B., Warkiani M.E. (2021). Simple-to-Operate Approach for Single Cell Analysis Using a Hydrophobic Surface and Nanosized Droplets. Anal. Chem..

[53] Xu L., Brito I.L., Alm E.J., Blainey P.C. (2016). Virtual Microfluidics for Digital Quantification and Single-Cell Sequencing. Nat. Methods.

[54] Zhou W.-M., Yan Y.-Y., Guo Q.-R., Ji H., Wang H., Xu T.-T., Makabel B., Pilarsky C., He G., Yu X.-Y. (2021). Microfluidics Applications for High-Throughput Single Cell Sequencing. J. Nanobiotechnol..

[55] Fung C.W., Chan S.N., Wu A.R. (2020). Microfluidic Single-Cell Analysis-Toward Integration and Total on-Chip Analysis. Biomicrofluidics.

[56] Collins D.J., Neild A., deMello A., Liu A.Q., Ai Y. (2015). The Poisson Distribution and beyond: Methods for Microfluidic Droplet Production and Single Cell Encapsulation. Lab Chip.

[57] Nguyen A., Khoo W.H., Moran I., Croucher P.I., Phan T.G. (2018). Single Cell RNA Sequencing of Rare Immune Cell Populations. Front. Immunol..

[58] Doud D.F.R., Bowers R.M., Schulz F., De Raad M., Deng K., Tarver A., Glasgow E., Vander Meulen K., Fox B., Deutsch S. (2019). Function-Driven Single-Cell Genomics Uncovers Cellulose-Degrading Bacteria from the Rare Biosphere. ISME J..

[59] Zimmerman S.B., Harrison B. (1987). Macromolecular Crowding Increases Binding of DNA Polymerase to DNA: An Adaptive Effect. Proc. Natl. Acad. Sci. USA.

[60] Minton A.P. (2001). The Influence of Macromolecular Crowding and Macromolecular Confinement on Biochemical Reactions in Physiological Media. J. Biol. Chem..

[61] Gaillard C., Strauss F. (1998). Avoiding Adsorption of DNA to Polypropylene Tubes and Denaturation of Short DNA Fragments. Tech. Tips Online.

[62] Belotserkovskii B.P., Johnston B.H., Gaillard C., Strauss F. (1996). Polypropylene Tube Surfaces May Induce Denaturation and Multimerization of DNA. Science.

[63] Kuznetsova I.M., Turoverov K.K., Uversky V.N. (2014). What Macromolecular Crowding Can Do to a Protein. Int. J. Mol. Sci..

[64] Ralston G.B. (1990). Effects of Crowding in Protein Solutions. J. Chem. Educ..

[65] Marcy Y., Ouverney C., Bik E.M., Lösekann T., Ivanova N., Martin H.G., Szeto E., Platt D., Hugenholtz P., Relman D.A. (2007). Dissecting Biological Dark Matter with Single-Cell Genetic Analysis of Rare and Uncultivated TM7 Microbes from the Human Mouth. Proc. Natl. Acad. Sci. USA.

[66] Labonté J.M., Field E.K., Lau M., Chivian D., Van Heerden E., Wommack K.E., Kieft T.L., Onstott T.C., Stepanauskas R. (2015). Single Cell Genomics Indicates Horizontal Gene Transfer and Viral Infections in a Deep Subsurface Firmicutes Population. Front. Microbiol..

[67] Bansal V. (2017). A Computational Method for Estimating the PCR Duplication Rate in DNA and RNA-Seq Experiments. BMC Bioinform..

[68] Zeileis A., Kleiber C., Rep M.A.Z.-T. (2014). 2009, U. Package “Ineq.”. https://cran.microsoft.com.

[69] Dorfman R. (1979). A Formula for the Gini Coefficient. Rev. Econ. Stat..

[70] Mikheenko A., Prjibelski A., Saveliev V., Antipov D., Gurevich A. (2018). Versatile Genome Assembly Evaluation with QUAST-LG. Bioinformatics.

[71] Jogia G., Tronser T., Popova A., Levkin P. (2016). Droplet Microarray Based on Superhydrophobic-Superhydrophilic Patterns for Single Cell Analysis. Microarrays.

[72] Feng W., Ueda E., Levkin P.A. (2018). Droplet Microarrays: From Surface Patterning to High-Throughput Applications. Adv. Mater..

[73] Chakraborty S., Luchena C., Elton J.J., Schilling M.P., Reischl M., Roux M., Levkin P.A., Popova A.A. (2022). “Cells-to-CDNA on Chip”: Phenotypic Assessment and Gene Expression Analysis from Live Cells in Nanoliter Volumes Using Droplet Microarrays. Adv. Healthc. Mater..

[74] Rinke C., Low S., Woodcroft B.J., Raina J.-B., Skarshewski A., Le X.H., Butler M.K., Stocker R., Seymour J., Tyson G.W. (2016). Validation of Picogram- and Femtogram-Input DNA Libraries for Microscale Metagenomics. PeerJ.

[75] Krueger F., James F., Ewels P., Afyounian E., Schuster-Boeckler B. Trim Galore. https://www.bioinformatics.babraham.ac.uk/projects/trim_galore/.

[76] Bushnell B. BBtools Software Package. https://sourceforge.net/projects/bbmap/.

[77] Wingett S.W., Andrews S. (2018). FastQ Screen: A Tool for Multi-Genome Mapping and Quality Control. F1000Research.

[78] Prjibelski A., Antipov D., Meleshko D., Lapidus A., Korobeynikov A. (2020). Using SPAdes De Novo Assembler. Curr. Protoc. Bioinform..

[79] R Core Team (2020). R: A Language and Environment for Statistical Computing. R Foundation for Statistical Computing; Vienna, Austria. https://www.R-project.org/.

[80] Villanueva R.A.M., Chen Z.J. (2019). Ggplot2: Elegant Graphics for Data Analysis (2nd Ed.). Measurement.

